# A boosting approach for prediction of protein-RNA binding residues

**DOI:** 10.1186/s12859-017-1879-2

**Published:** 2017-12-01

**Authors:** Yongjun Tang, Diwei Liu, Zixiang Wang, Ting Wen, Lei Deng

**Affiliations:** 1 Department of Clinical Pharmacology, Xiangya Hospital, Central South University, 87 Xiangya Road, Changsha, 410008 China; 2 Institute of Clinical Pharmacology, Hunan Key Laboratory of Pharmacogenetics, Central South University, 87 Xiangya Road, Changsha, 410008 China; 3 Department of Pediatrics, Xiangya Hospital, Central South University, 87 Xiangya Road, Changsha, 410008 China; 4 School of Software, Central South University, No.22 Shaoshan South Road, Changsha, 410075 China

**Keywords:** RNA-binding residue, Gradient tree boosting, Structural neighborhood features

## Abstract

**Background:**

RNA binding proteins play important roles in post-transcriptional RNA processing and transcriptional regulation. Distinguishing the RNA-binding residues in proteins is crucial for understanding how protein and RNA recognize each other and function together as a complex.

**Results:**

We propose PredRBR, an effectively computational approach to predict RNA-binding residues. PredRBR is built with gradient tree boosting and an optimal feature set selected from a large number of sequence and structure characteristics and two categories of structural neighborhood properties. In cross-validation experiments on the RBP170 data set show that PredRBR achieves an overall accuracy of 0.84, a sensitivity of 0.85, MCC of 0.55 and AUC of 0.92, which are significantly better than that of other widely used machine learning algorithms such as Support Vector Machine, Random Forest, and Adaboost. We further calculate the feature importance of different feature categories and find that structural neighborhood characteristics are critical in the recognization of RNA binding residues. Also, PredRBR yields significantly better prediction accuracy on an independent test set (RBP101) in comparison with other state-of-the-art methods.

**Conclusions:**

The superior performance over existing RNA-binding residue prediction methods indicates the importance of the gradient tree boosting algorithm combined with the optimal selected features.

## Background

Proteins binding with RNA through specific residues have a profound effect on many biological processes such as protein synthesis [1], post-transcriptional modifications, and regulation of gene expression [2–4]. Determining these protein-RNA binding residues can help to elucidate the underlying mechanisms, to control biological processes, or to design RNA-based drug. Some experimental techniques such as X-ray crystallography, NMR Spectroscopy and cross-linking approaches, have applied to investigate protein-RNA interface properties. However, large-scale experiments are expensive and difficult to carry out. Developing computational methods to predict RNA-binding sites precisely is becoming increasingly important.

In recent years, sequence and structural properties of protein-RNA binding residues have been widely analyzed and investigated [5]. A series of machine learning methods [6] such as Naive Bayes, support vector machine (SVM), and random forest (RF), combined with amino acid sequence or protein three-dimensional structural characteristics [4, 7], have been proposed to identify RNA-binding residues. Jeong et al. [8] build a neural network classifier to predict RNA-binding residues based on protein sequence and structural information. Wang and Brown [9] develop BindN, an efficient online approach that uses amino acid sequence and SVM to predict potential RNA-binding sites. Terribilini et al. [10, 11] propose a Naive Bayes classifier named RNABindR that can predict RNA-binding amino acids from 3D protein structures or protein sequences of unknown structure are most likely to interact with RNA. Liu et al. [12] implement a RF classifier to detect the RNA binding residues in proteins by integrating interaction propensity with other sequence and structural features. Other RNA-binding site prediction methods include PRINTR [13], RNABindRPlus [14], RBScore [15], NBench [16] and SNBRFinder [17].

Although existing studies [7, 9–24] have made remarkable progress to explore the interfaces of protein-RNA interactions, there is still great room for improvement. First, precise biological properties for precisely recognizing RNA-binding sites are not fully uncovered; no single feature can effectively identify protein-RNA interaction residues. Second, the number of non-binding sites is much higher than that of RNA-binding residues, which yields the so-called imbalance problem. Also, the imbalanced data tends to cause over-fitting and poor prediction results. Thus, developing effective approaches to address these issues at both data and algorithmic levels, such as feature extraction and selection, re-sampling techniques and one-class learning, is a pressing need.

In this work, we propose a novel RNA-binding residue prediction method named PredRBR, which takes advantage of Friedman’s gradient tree boosting (GTB) [25–27] and optimal selected features. PredRBR uses the GTB algorithm to iteratively build multiple classification trees based on the 44 optimal features selected from a series of sequence and structural features, especially two categories of structural neighborhood properties. The promising results of cross-validation and independent test demonstrate the effectiveness of PredRBR.

## Methods

### Datasets

We use RBP170 (previously named as RBP199) [13] as the training data set. The proteins in RBP199 were obtained from the protein-RNA complexes in Protein Data Bank (PDB) [28] as of May 2010. PISCES [29] was used to remove proteins with < 30% sequence identity or structures with resolution worse than 3.5Å. Proteins with residues < 40 or RNA-binding residues < 3 or the binding RNA with nucleotides < 5 were further excluded. Since there are 9 complexes (3HUW, 3I1M, 3I1N, 3KIQ, 2IPY, 2J01, 2QBE, 2Z2Q, 3F1E) in PDB obsoleted, a total of 170 protein sequences are generated.

Another independent dataset (BPP101) is collected from PDB with deposition date from June 2010 to May 2014. Similar to RBP170, only non-redundant and high-quality RNA-binding proteins are selected (sequence identity < 30% and resolution better than 3.5 Å). We also use CD-HIT [30, 31] to remove proteins with sequence similarity >40% to all proteins in RBP170. Finally, 101 protein sequences are obtained from 90 RNA-binding complexes.

**Fig. 1:**
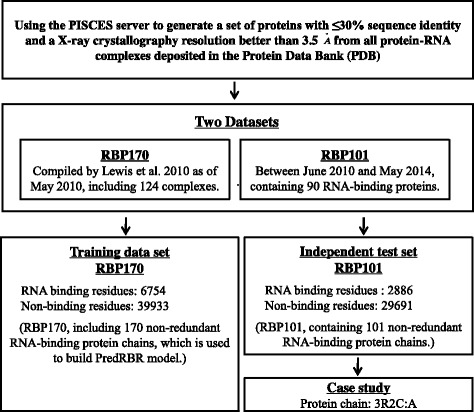
Summary of data set generation

**Fig. 2:**
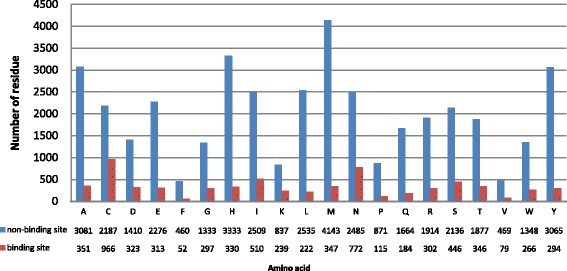
Number of RNA-binding and non-binding residues across the 20 amino acids in the RBP170 dataset

The two datasets are summarized in Fig. 1. A residue is defined as an RNA-binding site if there exists at least one atom in the protein with a distance cutoff < 5.0Å from an atom of the binding RNA [7, 9–11, 14–24]. RBP170 contains 6,754 (14.47%) RNA-binding sites and 39,933 (85.53%) non-binding sites. Figure 2 shows the distribution of RNA binding and non-binding residues across the 20 amino acids. BPP101 has 2886 RNA binding residues and 2,9691 Non-binding residues.

### Features extraction

A total of 63 sequence and structural site features (SiteFs) are calculated as follows:

**Physicochemical properties (10 features)**: The ten physicochemical properties are obtained from the AAindex database [32], including number of atoms, number of electrostatic charge, number of potential hydrogen bonds, molecular mass (Mmass), hydrophobicity, hydrophilicity, polarity, polarizability, propensities and average accessible surface area [33].

**Side-chain environment (pKa, 2 features)**: The side-chain environment pKa scores are extracted from Nelson and Cox [34] representing the side-chain environmental features of a protein.

**Position-specific scoring matrices(PSSMs, 20 features)**: PSSM profiles are quite effective in RNA-binding site prediction in previous studies [35–37]. We calculate PSSMs using PSI-BLAST [38] searching against the NCBI NR database, with iterations = 3 and *e*-value = 0.001.

**Evolutionary conservation score (C-score, 1 feature)**: We use Rata4Site [39] to calculate the C-score for each residue based on the sequence alignments.

**Solvent accessible area (ASA, 2 features)**: ASA properties are computed using DSSP [40], and the maximum solvent accessibility are calculated based on Rost and Sander [41].

**Secondary Structure (SS, 3 features)**: The secondary structure is also calculated using DSSP. The secondary structure can be divided into three categories: helix, sheet and coil. We encode the secondary structure as a 3-d vector. In the results of DSSP, types G, H and I are helix (1, 0, 0); types B and E are sheet (0, 1, 0); types T, S and blank are recognized as coil (0, 0, 1).

**Interaction propensity (IP, 4 features)**: Interaction propensity is first introduced by Liu [12]. The interaction propensity between the residue triplet *t* and the nucleotide *n* is defined as follows:

1 $$ IP(t, n) = \sum_{(P, R)} f_{(P, R)}(t, n) \log_{2}{\frac{f_{(P, R) }(t, n)}{f_{P}(t)f_{R}(n)}},  $$

where

2 $$ f_{(P, R)}(t, n) = \frac{N_{(P, R)}(t,n)}{\sum_{t, n} N_{(P, R) }(t, n)}  $$

3 $$ f_{P}(t) = \frac{N_{P} (t)}{\sum_{P}N_{P}(t)}  $$

4 $$ f_{R}(n) = \frac{N_{R} (t)}{\sum_{R}N_{R}(n)}  $$

In the above formulas, *f*_(*P*,*R*)_(*t*,*n*), *f*_*P*_(*t*) and *f*_*R*_(*n*) represent the frequency of amino acid triplet *t* that binds to nucleotide *n* in the protein-RNA pair (*P*,*R*), the frequency of triplet *t* in protein *P* and the frequency of nucleotide *n* in RNA *R*, respectively. *N*_(*P*,*R*)_(*t*,*n*) is the number of the amino acid triplet *t* interacting with nucleotide *n* in protein-RNA pair (*P*,*R*); $\sum _{t, n} N_{(P, R)} (t, n)$ is the total number of residue triplets that bind to any nucleotides in the protein-RNA pair (*P*,*R*); *N*_*P*_(*t*) is the number of triplet *t* in protein P; $\sum _{P}N_{P}(t)$ is the total number of amino acid triplets; *N*_*R*_(*n*) is the number of nucleotide *n* in RNA R and $\sum _{R }N_{R }(n)$ is the total number of nucleotides in the dataset. A total of 32,000 IPs are calculated for the 4 nucleotides and 20^3^ (8,000) residue triplets. For each residue, four features(*I* *P*_*A*_, *I* *P*_*U*_, *I* *P*_*G*_, *I* *P*_*C*_) are used to represent the interaction propensity (IP) of the residue triplet corresponding to different nucleotides (A, U, G and C).

**Disorder score (6 features)**: The disorder score is predicted using the method proposed by Obradovic et al. [42, 43].

**Atom contacts and residue contacts (2 features)**: We calculate the atom contacts (*N* *C*_*a*_) of an amino acid by aggregating all-atom contacts (*C*_*a*_) between the amino acid and any other residue in the protein, then dividing the number of atoms in the amino acid, as described in our previous work [44, 45]. Similarly, we compute the residue contacts (*N* *C*_*r*_) by summing all the contacts of the amino acid and then dividing the number of atoms in the amino acid.

**Pair potentials (PP, 1 feature)**: Contact potential (CP) between residue *i* and *j* is defined as follows:

5 $$  CP_{i, j}=\begin{cases} \ P_{i, j} \quad \ \text{if} \ | i-j | \geq 4 \ and \ d_{i, j} \leq 7 \text{\AA},\\ \ 0 \quad \ \text{otherwise}, \end{cases}  $$

where *P*_*i*,*j*_ is the contact potential of pair (i, j) collected from the work of Keskin et al. [46]; *d*_*i*,*j*_ is the distance between residue i and j. Note that the neighbors of a target residue are defined as a sphere of a certain radius of 7.0Å [47] based on the side chain center of mass. The overall contact potential of residue i (*P* *P*_*i*_) is calculated as follows:

6 $$ PP_{i} = \left | {\sum_{n=1}^{N}CP_{i, j}} \right | \ \ \ \ where \ \ |i - j| \geq 4  $$

**Topographical index (1 feature)**: The topographical score describes the structural environment of a amino acid. We compute the rate between structurally neighbor amino acids and the average number of residues for a specific amino acid type [44, 45, 48].

**Local structural entropy (LSE, 2 features)**: The local structural entropy [49] of a residue is calculated based on the protein sequence. The potential of a amino acid within a secondary structure (*β*-bridges, extended *β*-sheets, 3_10_-helices, *α*-helices, *π*-helices, bends, turns and other types) is estimated. More secondary structures the residue appeared in, the higher LSE score will be assigned. We compute the LSE score of a specific residue by averaging four successive sequence windows along the protein sequence. We also define a new attribute named *Δ*LSE to measure the difference of LSE value between the wild-type protein and its mutants.

**Four-body statistical pseudo-potential (FBS2P, 1 feature)**: The FBS2P score is based on the Delaunay tessellation of proteins [50], which can be calculated as a log-likelihood ratio:

7 $$ R_{ijmn}^{\alpha} = log\left[ {\frac{{f_{ijmn}^{\alpha} }}{{p_{ijmn}^{\alpha} }}} \right],  $$

where *i*, *j*, *m* and *n* are identities of the four amino acids (20 possibilities) in a Delaunay tetrahedron of the protein. Each point represents a residue. ${f_{ijmn}^{\alpha } }$ is the observed frequency of the residue composition (*ijmn*) in a tetrahedron of type *α* over a set of protein structures, while ${p_{ijmn}^{\alpha } }$ is the expected random frequency.

**Side chain energy score (SCE-score, 6 features)**: The SCE-score is a linear combination of multiple energetic terms, including surface area of atom binding, overlap volume, hydrogen bonding energy, electrostatic interaction energy, buried hydrophobic SAS area and buried SAS area between the target residue and the rest of the protein, respectively [50].

**Voronoi contacts (2 features)**: The Voronoi contact is calculated based on the Voronoi neighbors in protein structure, as described in Ref. [51].

**Structural Neighborhood Features (SNF-EDs & SNF-VDs)**: In this work, two types of structural neighborhood features (Euclidean and Voronoi) are used. This two structural neighborhood groups named as SNF-EDs and SNF-VDs are defined based on Euclidean distance and Voronoi division [44] respectively. The SNF-EDs is a set of residues located within a sphere of 10Å in Euclidean distances from the central residue. The feature *i* for a neighbor *n* (the n-th residue) with regard to the target residue *r* (the r-th residue) is defined as follows:

8 $$  {\begin{aligned} F_{i}(r, n)=\left\{ \begin{array}{ll} \text{the value of feature {i} for residue} & {r} \ \text{if} \ | r-n | \geq 1 \\ & and \ d_{r, n} \leq 10 {{\r{A}}}, \\ \ 0 \quad \text{otherwise}, \end{array}\right. \end{aligned}}  $$

where *d*_*r*,*n*_ is the minimum Euclidean distance between any heavy atoms of residue *r* and that of residue *n*. The SNF-EDs of target residue *r* is defined as:

9 $$ EN_{i}(r)=\sum_{n=1}^{m} F_{i}(r, n),   $$

where *m* is the total number of Euclidean neighbors.

We also use Voronoi division to define neighbor residues. For each protein 3D structure, the 3D space is partitioned into Voronoi polyhedra around individual atoms. A pair of residues are defined to be Voronoi neighbors when there exits a Voronoi facet in common for the two residues. The Qhull package [52] is used to compute Voronoi division.

Give the target residue *r* and its neighbors *n* {*n*=1,...,*m*}, for each site feature *i*, a Voronoi neighborhood property is defined as:

10 $$ VD_{i}=\sum_{n=1}^{m} P_{i}(n),  $$

where *P*_*i*_(*n*) is the value of the residue feature *i* for neighbor *n*.

Finally, a large number of 63×3=189 site, Euclidean and Voronoi characteristics [53] are obtained for RNA-binding site prediction.

### Gradient tree boosting algorithm

The Gradient Tree Boosting (GTB) [25–27] is an effective ensemble method for regression and classification issues. Here we apply GTB to predict RNA binding residues. For the input feature vectors *χ*_*i*_ (*χ*_*i*_={*x*_1_,*x*_2_,…,*x*_*n*_},*i*=1,2,…,*N*) with labels *y*_*i*_ (*y*_*i*_ *ε*{−1,+1},*i*=1,2,…,*N*, where “-1” denotes non-binding resides and “+1” represents RNA-binding sites. The details of the GTB algorithm is shown in Algorithm 1.



In this algorithm, the number of iterations is initialized as *M*; *L*(*y*,*Θ*(*x*)) is the log loss function; *y* represents the label and *Θ*(*χ*) is a decision function; *N* is the number of residues in RBP170. The GTB algorithm iteratively repeats steps 2-7 to build m different classification trees *h*(*χ*,*α*_1_),*h*(*χ*,*α*_2_),...,*h*(*χ*,*α*_*m*_) from a set of training data. *β*_*m*_ is the weight and *α*_*m*_ is the parameter vector of the *m*th tree *h*(*χ*,*α*_*m*_). At the end, we can obtain the function *Θ*_*M*_(*χ*) and build a GTB model $\tilde {\Theta }(\boldsymbol{\chi })$. Note that the GTB algorithm is implemented using scikit-learn [54].

### The PredRBR framework

**Fig. 3:**
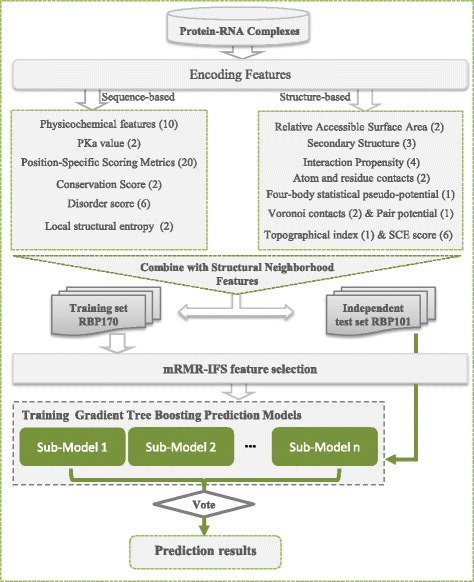
Flowchart of PredRBR. A total of 189 sequence and structure-based features including two categories of Euclidean and Voronoi neighborhood features are obtained. Then we use the mRMR-IFS approach to select an optimal set of 177 properties. Finally, we use the Gradient Tree Boosting algorithm and the balanced under-sampling techniques to build the RNA-binding site prediction models

The flow chart of PredRBR is shown in Fig. 3. A wide range of sequence and structural site features (63 SiteFs), and two groups of neighborhood attributes (63 SNF-EDs and 63 SNF-VDs) are computed. We use the Maximum Relevance Minimum Redundancy and Incremental Feature Selection (mRMR-IFS) [55] approach to select a small subset of optimal features that make the greatest contribution to the classification.

**maximum Relevance Minimum Redundancy (mRMR)** mRMR means that a feature may be selected preferentially has the maximal correlation with the target attribute and minimal redundancy with the characteristics already chosen. mRMR is measured with mutual information (MI), and the definition is as follows:

11 $$ I(x, y) - \iint p(x, y) log \frac{p(x,y)}{p(x)p(y)}dxdy,  $$

where *x* and *y* are two random attributes; *p*(*x*,*y*) is the joint probabilistic density; *p*(*x*) and *p*(*y*) are the marginal probabilistic densities. The detailed description of mRMR can be found in Ref. [55]. An ordered list of features are obtained by applying mRMR to the benchmark RBP170 with 189 features.

**Incremental Feature Selection (IFS)** Based on the ordered feature list generated by mRMR, we use IFS to decide the optimal feature set. A total number of *n* feature sets are generated based on the mRMR results as follows:

12 $$ F_{i} = \{f_{1}, f_{2}, \ldots, f_{i}\} \ \ (1\leqslant i\leqslant n),  $$

where *f*_*i*_ is the *i*−*t* *h* sorted feature; *F*_*i*_ is the *i*−*t* *h* feature set; *n* is the number of features. We use the GTB algorithm to build classifiers based on each feature subset *F*_*i*_ and evaluate the performance with 10-fold cross-validation. We select the feature subset with the highest overall performance (AUC+MCC) as the optimal feature set.

**Deal with the imbalance problem** In the benchmark RBP170, the amount of non-binding sites is about 6 times that of RNA binding sites. To deal with the imbalance problem, we use a random under-sampling strategy to generate the new balanced datasets. In the training set, negative samples (non-binding sites) are randomly selected and combined with the positive samples create a 1:1 balance dataset.

### Evaluation measures

To evaluate the performance of PredRBR, some widely used measurements are also adopted, including sensitivity (SN/Recall), specificity (SP), precision (Pre), accuracy (ACC), F-measure and Matthews Correlation Coefficient (MCC) score. These metrics are defined as follows:

13 $$ SN (Recall)= \frac { TP }{ TP + FN }  $$

14 $$ SP = \frac { TN }{ TN + FP}  $$

15 $$ Precision = \frac { TP }{ TP + FP }  $$

16 $$ ACC = \frac { TP + TN }{ TP + TN + FP + FN }  $$

17 $$ F-measure = \frac{2 \times Recall \times Precision}{ Recall + Precision}  $$

18 $$  MCC = \frac{TP \times TN - FP \times FN}{ \sqrt {(TP + FP)(TP + FN)(TN + FP)(TN+FN)}}  $$

In these equations, the TP, TN, FP, FN refer to the numbers of true positive, true negative, false positive and false negative residues in the prediction, correspondingly. In addition, the ROC graph is formed by plotting the false positive rate (i.e. 1 - specificity) against the true positive rate, which equals sensitivity. Furthermore, the area under the receiver operating characteristic (ROC) [56] curve (AUC) is also utilized for evaluating prediction performance.

## Results and discussion

In this section, we first tested the prediction performance of the PredRBR model with different combinations of features, including PSSMs, site features (SiteFs) and structural neighborhood features (SNF-EDs & SNF-VDs), and compared the performance of SiteFs and structural neighborhood features. Then, the mRMR-IFS method is used to select the optimal feature set from all obtained properties. We also implemented many machine learning algorithms using the selected features and compared the prediction performance of gradient tree boosting classifier with these methods using 10-fold cross-validation. Finally, we compared the PredRBR model with existed previous approaches on the same independent test set, and an example of the predicted interface residues with RNA in the protein 3R2C:A is provided to illustrate the proposed method.

### Evaluation of different feature combinations

In previous approaches, many combinations of features have been widely applied to get improved predictions of protein-RNA interaction residues, including physicochemical features, side-chain environment, sequence conservation score, position-specific scoring matrices (PSSMs), relative accessible surface area (RASA), secondary structure (SS), interaction propensity and so on. Based on these researches [7, 9–11, 14–24], we combined a variety of features of the amino acids to represent the specific interaction attributes of protein residues with RNA nucleotides. In this work, some of the site characteristics, such as relative accessible surface area, secondary structure and interaction propensity, can be calculated only after the protein structure information is available. Thus, we categorize these site features into structure-based characteristics, and others are sequence features. To investigate the performances of different features combinations, including the mRMR-IFS selected features, we build a series of sub-models based on the those features and compared the prediction performances of these model using 10-fold cross-validation on the RBP170 dataset. The detailed results are depicted in Table 1. The performance of each model is measured by seven metrics: accuracy (ACC), sensitivity (SN), specificity (SP), Precision, F-measure, MCC and area under curve (AUC). Note that the site features (SiteFs) is the 63D basic sequence and structure properties, including none of structural neighborhood features, and the PSSM column in Table 1 is a subset of the site features.

**Table 1:** The cross-validation results of different feature combinations and the optimal selected feature set using mRMR-IFS on the RBP170 dataset

Features	ACC	SN	SP	Precision	F-measure	MCC	AUC
PSSM	0.72 ± 0.01	0.69 ± 0.02	0.73 ± 0.01	0.30 ± 0.01	0.42 ± 0.02	0.31 ± 0.02	0.79 ± 0.01
SiteFs	0.77 ± 0.01	0.74 ± 0.02	0.77 ± 0.01	0.36 ± 0.01	0.48 ± 0.01	0.40 ± 0.02	0.84 ± 0.01
SNF-VDs	0.75 ± 0.01	0.80 ± 0.01	0.74 ± 0.01	0.35 ± 0.02	0.48 ± 0.02	0.40 ± 0.02	0.85 ± 0.01
SNF-EDs	0.78 ± 0.01	0.79 ± 0.02	0.78 ± 0.01	0.38 ± 0.02	0.51 ± 0.01	0.44 ± 0.02	0.87 ± 0.01
SNF-EDs+SNF-VDs	0.82 ± 0.01	0.81 ± 0.02	0.82 ± 0.01	0.44 ± 0.02	0.57 ± 0.02	0.51 ± 0.02	0.89 ± 0.01
SiteFs+SNF-EDs+SNF-VDs	0.82 ± 0.01	0.83 ± 0.01	0.83 ± 0.01	0.46 ± 0.02	0.58 ± 0.01	0.53 ± 0.01	0.91 ± 0.01
mRMR-IFS (Top177)	0.84 ± 0.01	0.85 ± 0.02	0.84 ± 0.01	0.47 ± 0.02	0.60 ± 0.02	0.55 ± 0.02	0.92 ± 0.01

As shown in Table 1, the performance of prediction based on PSSM is not so good, at least not reach our research aims. In contrast, the method with site features (SiteFs) achieves a relatively good performance with a AUC value of 0.84, there is at least 5% increase in overall accuracy, sensitivity, specificity, MCC, F-measure and AUC score compared with PSSM. The Euclidean neighborhood features (SNF-EDs) outperforms PSSM and SiteFs, with at least a 3% improvement on AUC score, which suggests that SNF-EDs is an important feature type for predicting protein-RNA binding residues. When combining all of the structural neighborhood features (SNF-EDs+SNF-VDs), the improvement on performance is impressive, at least 4% increase in ACC and 5% increase in AUC score compared with site features (SiteFs). The optimal 177 features (Top177) are selected from the full combined features (SiteFs+SNF-EDs+SNF-VDs) with an effective feature selection method (mRMR-IFS [55]) and achieve the best performance.

### Contribution of feature selection

**Fig. 4:**
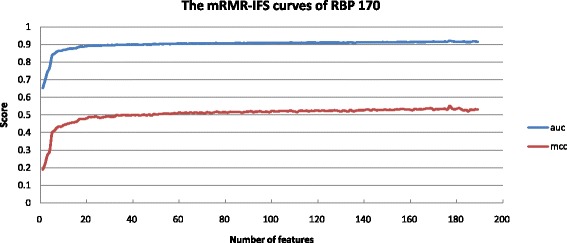
The performance (AUC and MCC) of the top *N* features using the mRMR-IFS approach

Selecting the most informative features is essential for the prediction performance enhancement, and may consequently improve our understanding of the molecular mechanism of RNA-binding sites. A total of 189 site, Euclidean and Voronoi features are initially calculated. We use mRMR-IFS [55], a filter-based approach to rank the features and select the top *k* attributes. The classifier with the top 177 features achieves the highest performance (MCC = 0.55 and AUC = 0.92) in cross-validation on RBP170 (Fig. 4). We select the 177 optimal features to build the final RNA-binding site prediction model. As shown in Table 1, the performance of the top 177 features selected using mRMR-IFS is significantly better than that of other feature combinations.

**Fig. 5:**
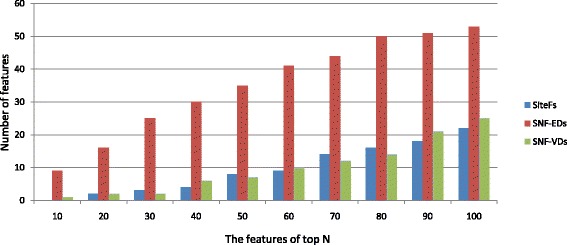
The numbers of different feature categories existing in the top *N* ordered features

We also analyze the numbers of sits (SiteFs), Euclidean (SNF-EDs) and Voronoi (SNF-VDs) features that occurred in the top *N* characteristics sorted by using the mRMR method, respectively. Figure 5 shows the numbers of the three categories of features exited in the top *N* (range from 10 to 100) selected properties. We observed that structural neighborhood characteristics (SNF-EDs and SNF-VDs) [44] occupy the majority of the top *N* list, implying that structural neighborhood characteristics paly a critical role in boosting the performance of RNA-binding residue prediction.

### Performance comparison with other machine learning methods

We further compare the effectiveness of PredRBR with existing state-of-the-art machine learning methods, including Support Vector Machine (SVM) [57], Random Forest (RF) [58] and Adaboost [59]. Table 2 shows the prediction results of these classifiers. It is worth indicating that all examined methods employ the same feature set on the training dataset (RBP170) with 10-fold cross-validation. With a specificity of 0.84, PredRBR obtains a sensitivity of 0.85, a precision of 0.47, a F-measure of 0.60 and a MCC value of 0.55. The best one among these compared machine learning methods is Random Forest with its sensitivity of 0.81 and specificity of 0.83 as well as F-measure of 0.57. Comparing with Random Forest, PredRBR obtains at least 2% increase in sensitivity, 7% increase in MCC value and 5% increase in F-measure. PredRBR also achieves higher AUC score than that of other comparison machine learning approaches. The AUC score of PredRBR is 0.92, while those of the three machine learning methods are in the range of 0.87 ∼0.90. The results imply that our proposed GTB-based PredRBR model plays crucial role in performance boosting.

**Table 2:** Prediction performance of PredRBR and other machine learning methods on the RBP170 dataset

Method	ACC	SN	SP	Precision	F-measure	MCC	AUC
PredRBR	0.84 ± 0.01	0.85 ± 0.02	0.84 ± 0.01	0.47 ± 0.02	0.60 ± 0.02	0.55 ± 0.02	0.92 ± 0.01
RF	0.82 ± 0.01	0.81 ± 0.01	0.83 ± 0.01	0.44 ± 0.02	0.57 ± 0.02	0.51 ± 0.02	0.90 ± 0.01
SVM	0.81 ± 0.01	0.81 ± 0.02	0.81 ± 0.02	0.42 ± 0.01	0.55 ± 0.01	0.49 ± 0.01	0.89 ± 0.01
Adaboost	0.79 ± 0.01	0.80 ± 0.01	0.79 ± 0.01	0.40 ± 0.01	0.53 ± 0.01	0.46 ± 0.01	0.87 ± 0.01

### Results of the independent evaluation

**Fig. 6:**
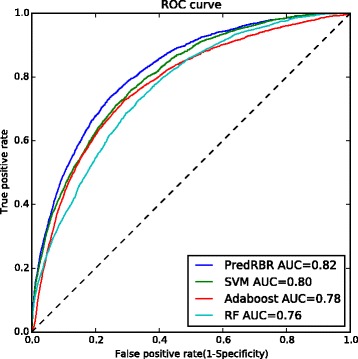
The ROC curves of PredRBR and other three machine learning methods on the RBP101 dataset

We validate the usability of the proposed PredRBR model on the independent test dataset. The independent test dataset (RBP101) has 101 non-homologous proteins including 2886 binding sites and 29704 non-binding sites. Due to the imbalance between positive sample and negative sample, the receiver operating characteristic (ROC) curve is regarded as proper measurement to evaluate the overall performance. Higher curve of ROC represents better prediction accuracy. Figure 6 shows the ROC curves and AUC scores of PredRBR and other machine learning methods on the RBP101 dataset. PredRBR, SVM, Adaboost and Random Forest achieve AUC values of 0.82, 0.80, 0.78 and 0.76, respectively. Comparing with the other methods, the PredRBR model improves the AUC score by 2% ∼6%.

We compare PredRBR with several existing state-of-the-art RNA-binding residue prediction approaches, including BindN [9], PPRint [20], Liu-2010 [12], BindN+ [22], RNABindR2.0 [23], RNABindRPlus [14] and SNBRFinder [17] on the independent set (RBR101). In these methods, BindN [9], BindN+ [9] and PPRint [20] use SVM to build the RNA-binding site classifier; RNABindRPlus [14] utilizes a logistic regression method to integrate the homology-based method HomPRIP and optimized SVM model named SVMOpt; Liu-2010[12] is RF-based method with sequence and structural features especially the proposed interaction propensity, and SNBRFinder [17] is a hybrid method based on the sequence features.

**Fig. 7:**
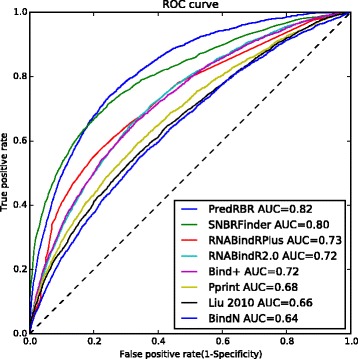
The ROC curves of PredRBR and other state-of-the-art prediction approaches on the RBP101 dataset

As shown in Table 3, PredRBR achieves the best predictive performance with an accuracy of 0.83, a sensitivity of 0.59, specificity of 0.85, precision of 0.28, F-measure of 0.38 and MCC of 0.32. The results indicate that 59% of the real RNA-binding residues are correctly identified (sensitivity), and 85% of the non-RNA binding residues are precisely predicted (specificity). In the control methods, SNBRFinder gains the best prediction results (sensitivity=0.65, specificity=0.80, F-measure=0.36 and MCC=0.31). The performance our PredRBR method goes beyond SNBRFinder regarding F-measure and MCC. Particularly, the specificity of PredRBR is significantly better than that of RNABindR (increased by 5%), which suggests that PredRBR would be able to determine the residues that do not exist in the RNA-binding surface better and reduce the experiment cost. The ROC curves of PredRBR and other existing methods are shown in Fig. 7, which are drawn by varying the cutoffs of the prediction scores to calculate the sensitivities and specificities of these methods. The AUC scores (areas under ROC curves) of the eight methods, including PredRBR, SNBRFinder, RNABindRPlus, RNABindR 2.0, BindN+, PPRint, Liu-2010, BindN, are about 0.82, 0.80, 0.73, 0.72, 0.72, 0.68, 0.66 and 0.64, respectively. These improvements on the prediction indicate that our proposed PredRBR method integrating the GTB algorithm and the optimal selected 177 features particularly the structural neighborhood properties can effctively predict RNA-binding residues.

**Table 3:** Independent test of our GTB-based PredRBR and other existing methods on the RBP101 dataset

Method	ACC	SN	SP	Precision	F-measure	MCC
PredRBR	0.83 ± 0.12	0.59 ± 0.13	0.85 ± 0.11	0.28 ± 0.16	0.38 ± 0.15	0.32 ± 0.17
SNBRFinder	0.78 ± 0.15	0.65 ± 0.22	0.80 ± 0.13	0.25 ± 0.21	0.36 ± 0.18	0.31 ± 0.20
RNABindRPlus	0.80 ± 0.10	0.49 ± 0.30	0.84 ± 0.13	0.26 ± 0.26	0.34 ± 0.24	0.26 ± 0.22
BindN+	0.81 ± 0.09	0.42 ± 0.18	0.85 ± 0.05	0.22 ± 0.24	0.29 ± 0.18	0.21 ± 0.17
RNABindR 2.0	0.71 ± 0.09	0.59 ± 0.22	0.72 ± 0.14	0.17 ± 0.16	0.27 ± 0.16	0.20 ± 0.12
PPRint	0.82 ± 0.09	0.35 ± 0.19	0.86 ± 0.06	0.20 ± 0.27	0.25 ± 0.17	0.17 ± 0.15
Liu-2010	0.73 ± 0.07	0.51 ± 0.19	0.72 ± 0.10	0.15 ± 0.14	0.23 ± 0.15	0.15 ± 0.11
BindN	0.69 ± 0.07	0.49 ± 0.15	0.70 ± 0.05	0.14 ± 0.20	0.22 ± 0.15	0.12 ± 0.13

### Case study

**Fig. 8:**
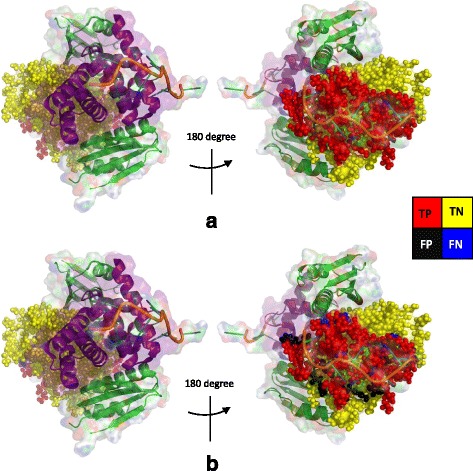
Comparison between experimentally determined RNA binding sites (**a**) and predicted RNA binding residues (**b**). **a** Actual RNA-binding residues in protein 3R2C:A. Result of (**b**) is predicted binding residues by PredRBR and the numbers of predicted TP, FP, TN and FN in 3R2C:A are 30, 8, 92, and 8, respectively. The true positive, true negative, false positive and false negative residues are shown in red, yellow, black and blue, respectively

The ternary NusB-NusE-BoxA RNA complex (PDB code 3R2C) initiates the complete antitermination complex required by the processive transcription antitermination. The complex NusB-NusE-BoxA reveals the significance of key protein-protein and protein-RNA interactions. Here, we use PredRBR to investigate the RNA binding residues in NusB (3R2C:A). The overall accuracy of predicting RNA binding residues by PredRBR is 0.88, which is a very accurate when compared with the available experimental data. Figure 8 shows the comparison between actual interaction residues and predicted RNA binding residues in the protein 3R2C:A. Figure 8a presents the actual interaction residues of protein 3R2C:A and the red spheres represent real RNA binding residues. Figure 8b shows the binding sites predicted by PredRBR. The results show that most of the actual interaction residues are well identified by the PredRBR model.

## Conclusion

In this study, we have developed PredRBR, a high-performance protein-RNA binding site prediction method. The novelty of the proposed method lies in the idea that we widely integrate a large number of sequence, structural and energetic characteristics, together with two categories of Euclidian and Voronoi neighborhood features, produces more critical clues for RNA-binding residue prediction. A total of 63 site-based, 63 Euclidian and 63 Voronoi neighborhood features have been obtained. We use the mRMR-IFS approach to select an optimal subset of 177 features to reduce the computational time and improve the performance. Our results also highlight the benefits of basing RNA-binding residue prediction method on the GTB algorithm and structural neighborhood characteristics (Euclidian and Voronoi). Both cross-validation and independent test show that PredRBR performs significantly better than other existing state-of-the-art methods such as Liu-2010, BindN+, RNABindRPlus, BindN, PPRint, SNBRFinder and RNABindR2.0. Furthermore, we demonstrate the effectiveness of our approach to an RNA binding complex and obtained encouraging results.

A limitation of PredRBR is that it is a structure-based approach, which use an encoding of sequence and structure-derived features of a target residue and its structural neighborhood features to make predictions. RNA-binding sites of proteins without known 3D structures can’t be well predicted. However, the number of proteins with known structures has increased rapidly in the past few years especially due to the accurate theoretical models that can be produced when using the solved representatives as templates for the models.

In the future, we will try to extract more effective features and machine learning methods to further improve the RNA-binding residue prediction. Also, we will develop an open access web-server for the proposed PredRBR method.
